# Common *interleukin-6 *promoter variants associate with the more severe forms of distal interphalangeal osteoarthritis

**DOI:** 10.1186/ar2374

**Published:** 2008-02-08

**Authors:** Olli-Pekka Kämäräinen, Svetlana Solovieva, Tapio Vehmas, Katariina Luoma, Hilkka Riihimäki, Leena Ala-Kokko, Minna Männikkö, Päivi Leino-Arjas

**Affiliations:** 1 Collagen Research Unit, Biocenter and Department of Medical Biochemistry and Molecular Biology, University of Oulu, 90220 Oulu, Finland; 2 Centre of Expertise for Health and Work Ability, Finnish Institute of Occupational Health, 00250 Helsinki, Finland; 3 Department of Radiology, Helsinki University Central Hospital, 00290 Helsinki, Finland; 4 Connective Tissue Gene Tests, Allentown, PA 18103, USA

## Abstract

**Introduction:**

The objective of this study was to investigate the relationship of the *IL-6 *promoter variants G-597A, G-572C and G-174C (rs1800797, rs1800796 and rs1800795, respectively), which have been shown to affect both the transcription and secretion of IL-6, to symptomatic distal interphalangeal (DIP) osteoarthritis (OA).

**Methods:**

A total of 535 women aged 45 to 63 years were included. Radiographs of both hands were taken and each DIP joint was evaluated (grade 0 to 4) for the presence of OA. Information on symptoms (pain, tenderness) in each joint was collected by using a self-administered questionnaire. Symptomatic DIP OA was defined by the presence of both radiographic findings of grade 2 or more and symptoms in at least two DIP joints, and symmetrical DIP OA by the presence of radiographic findings of grade 2 or more in at least one symmetrical pair of DIP joints. Common polymorphic loci in the *IL-6 *gene were amplified and the promoter haplotypes were reconstructed from genotype data with the PHASE program. Logistic regression analysis was used to examine the association between the *IL-6 *genotypes/diplotypes and the DIP OA outcome.

**Results:**

The G alleles of two promoter single nucleotide polymorphisms (SNPs) G-597A and G-174C were more common among the subjects with symptomatic DIP OA than among those with no disease (*P *= 0.020 and 0.024, corrected for multiple testing). In addition, the carriage of at least one G allele in these positions increased the risk of disease (*P *= 0.006 and *P *= 0.008, respectively). Carrying a haplotype with the G allele in all three promoter SNPs increased the risk of symptomatic DIP OA more than fourfold (odds ratio (OR) 4.45, *P *= 0.001). Carriage of the G-G diplotype indicated an increased risk of both symmetrical DIP OA (OR 1.52, 95% confidence interval 1.01 to 2.28) and symptomatic DIP OA (OR 3.67, 95% confidence interval 1.50 to 9.00).

**Conclusion:**

The present study showed that the presence of G alleles at common *IL-6 *polymorphic promoter loci was associated with the more severe DIP OA outcomes, symmetrical and symptomatic.

## Introduction

Osteoarthritis (OA) is a degenerative disorder of the synovial joints causing pain and premature wear and loss of articular cartilage. It is the most common form of arthritic disease, with a strong genetic component [1-3]. The genetic etiology of this complex disease is not well known, although genome-wide linkage analyses and individual gene studies have recently uncovered several genomic areas containing OA-associated variants [4]. The joints of the hand are most commonly affected by OA [5], and hand OA is highly prevalent particularly among middle-aged women, often being polyarticular [6]. Furthermore, it has been demonstrated recently that the genetic determinants of OA are sex-related and that a joint-specific approach to the genetics of this condition may be more rewarding than a global approach [7,8]. OA of the distal interphalangeal (DIP) joints is a homogeneous form of OA [9,10] that has yielded positive results in genome scans, which has previously been unsuccessful in studies that have treated hand OA as a single entity [8,11].

IL-6 is believed to be one of the major factors in joint destruction, being a pleiotropic pro-inflammatory cytokine that is markedly upregulated at times of tissue inflammation. A significant increase in the level of IL-6 mRNA has been detected in OA-affected cartilage, and the IL-6 levels in the serum and synovial fluid have been reported to be elevated among OA patients [12]. In addition, human recombinant IL-6 has been shown to enhance human recombinant IL-1β-induced proteoglycan degradation and to inhibit chondrocyte proliferation [13]. Known variations within the *IL-6 *gene have been repeatedly screened in various association studies. According to the reports, a common guanine/cytosine polymorphism at position -174 in the promoter region of the *IL-6 *gene seems to have a role in a variety of diseases and conditions [14]. This variation regulates the transcription of the *IL-6 *gene and is associated with plasma levels of IL-6 [15]. The activity of the promoter is also affected by the nearby polymorphic sites at -597 and -572, which seem to control the influence of the polymorphism at position -174 [16].

Previous studies have suggested that the allelic variations and common haplotypes of the *IL-6 *gene are related to cartilage-degrading conditions [17,18]. Functional experiments to study polymorphic effects on the synthesis of IL-6 have shown that its transcription and synthesis are affected by the allelic variations within the gene, although the mechanism and level of the contribution to the actual cartilage-degrading process is still under debate. Another point of view is that IL-6 molecules seem to contribute to the development of the pain sensation and that this effect can be modified by genomic variations, especially in the promoter area of the gene [19]. IL-6 is markedly upregulated in various pathologic situations generally associated with pain and hyperalgesia [20], and its administration on the skin provokes pain, which increases if it is injected into the cerebrospinal fluid [21].

This study was undertaken to define how strongly the common genetic variations within the *IL-6 *gene contribute to the different forms of DIP OA. We report here that the presence of G alleles at common *IL-6 *polymorphic promoter loci was associated with the more severe forms of DIP OA, symptomatic and symmetrical, in our sample.

## Materials and methods

### Subjects

Potential subjects representing two occupational groups with a similar socio-economic status but completely different hand loads, namely dentistry and teaching, were identified through the Finnish Dental Association and the Finnish Teachers' Union. Four hundred and thirty-six women aged 45 to 63 years were randomly selected from each occupational group, using the place of residence as an inclusion criterion (Helsinki or its neighboring cities) for participation in a study concerning work-related factors and individual susceptibility in the etiology of hand osteoarthritis. Of those who received the questionnaires, 295 dentists (67.7%) and 248 teachers (56.9%) participated in a clinical examination. This participation was voluntary, and signed informed consent was obtained from all subjects. The study was approved by the local ethics committee for research in occupational health and safety.

### Clinical and radiological assessments

Radiographs of both hands were taken for all the participants, and each DIP joint was evaluated and analyzed by an experienced radiologist who was blinded to all the data regarding the subjects. The presence of hand OA was defined by using a modified Kellgren and Lawrence system [22] based on the following criteria: grade 0 = no OA (normal finding); grade 1 = suspected OA; grade 2 = mild OA; grade 3 = moderate OA; grade 4 = severe OA. Reference images, as described elsewhere [6], were used. A second reading was performed independently by the original radiologist and another radiologist for 46 randomly selected subjects. The intra-observer and inter-observer agreements [23] indicated good reliability for the readings and the grading of OA (from 0.73 to 0.88 and from 0.67 to 0.85, respectively) [6].

If the subject had at least two DIP joints with radiographic OA of grade 2 to 4, she was classified as having finger DIP OA. Symmetrical DIP OA was defined as a subcategory of DIP OA: OA in at least one symmetrical pair of the DIP joints (if one DIP joint of the hand is affected, the same joint of the opposite hand is also affected).

Information on symptoms (pain, tenderness) in each joint studied was collected by means of the self-administered questionnaire, with the prompt: 'Please point out on the picture below in which finger joint you have felt pain or tenderness during the past 30 days.' The subjects were also asked to mark the intensity of the pain: 0 = no pain, 1 = mild pain, 2 = moderate pain, 3 = severe pain. If the subject had both radiographic findings (grade ≥ 2) and symptoms (grade ≥ 1) in at least two corresponding DIP joints, she was classified as having symptomatic DIP OA.

Data regarding individual characteristics were collected by means of a self-administered questionnaire, which included items on anthropometric measures and smoking history. Weight was measured without shoes to an accuracy of 0.1 kg. Body mass index (BMI) (weight (kg) divided by height squared (m^2^)) was calculated on the basis of the subject's self-reported height and the weight as measured at the clinical examination. BMI was divided into tertiles for logistic regression analysis (low, less than 22.5 kg/m^2^; medium, 22.5 to 25.5 kg/m^2^; high, more than 25.5 kg/m^2^). In terms of their smoking history, the subjects were classified into those who had never smoked and those who had smoked at some time (current or previous smokers).

### Genotyping of the IL-6 genomic variants

Three sites with a single nucleotide polymorphism (SNP) at the promoter positions -597, -572 and -174 (G-597A, G-572C and G-174C) were investigated. The corresponding SNP reference numbers are rs1800797, rs1800796 and rs1800795. Genomic DNA was prepared from EDTA-anti-coagulated peripheral blood and used as a template for PCR. The DNA was amplified in a total reaction volume of 23 μl. Genomic DNA (20 ng) was used with 0.25 μM forward and reverse primers, 1.5 μM MgCl_2_, 0.2 mM dNTPs and 1 U of Ampli Tag Gold polymerase (Applied Biosystems, Foster City, CA, USA). Polymorphic loci were amplified by using previously described primer sequences [17]. PCR cycling conditions were as follows: initial denaturation for 10 minutes at 95°C, 35 cycles at 95°C for 30 s, 60 to 68°C for 30 s and 72°C for 30 s, and a final extension at 72°C for 10 minutes. All the PCR products were sequenced with an ABI PRISM™ 3100 sequencer and BigDye Terminator sequencing kit (Applied Biosystems) to obtain adequate definition of the genotype for all subjects with respect to the different polymorphic loci. The genotyping and recording of the results took place in a double-blinded manner without any personal information on the subjects, for example age or occupation.

For technical reasons, eight blood samples were not genotyped. Of the 535 samples analyzed, we failed to obtain a genotype at promoter position -174 for two subjects and at positions -597 and -572 for three subjects.

### Statistical methods

**Figure 1:**
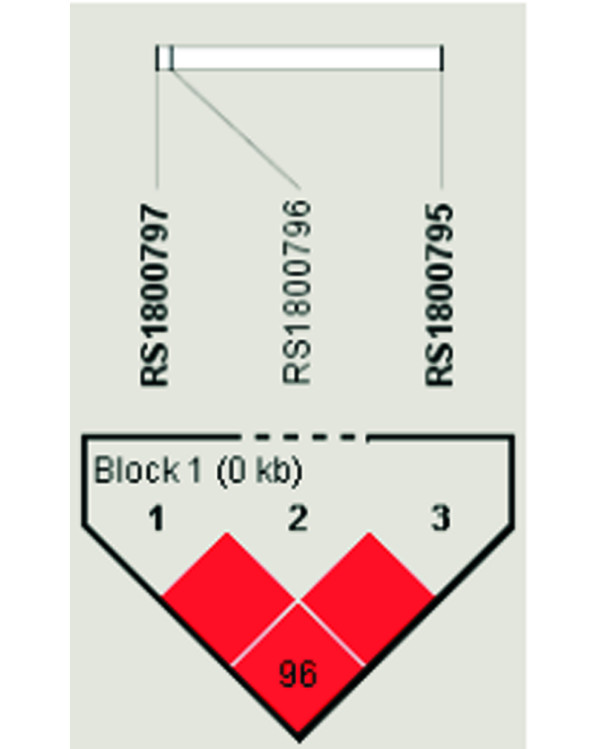
Haploview linkage disequilibrium plot of the *IL-6 *promoter single nucleotide polymorphisms rs1800797, rs1800796 and rs1800795.

Student's *t *test or the χ^2 ^test was used to compare individual characteristics between subjects with and without symptomatic DIP OA. Potential deviation from Hardy–Weinberg equilibrium was tested with the χ^2 ^test. Fisher's exact probability test or the χ^2 ^test was used to compare allele and genotype frequencies between individuals with and without symptomatic DIP OA. The allelic association of each locus was first investigated separately, and corrected *P *values (*P*_corr_) were calculated by multiplying *P *by the number of alleles compared. Because the loci are in close proximity to each other, haplotype analysis was performed to investigate whether underlying linkage disequilibrium (LD) contributed to the non-independence of these associations. The degree of pairwise LD was calculated for each pair of SNPs with the Haploview software [24]. An LD plot for the SNPs studied here is presented in Figure 1 and the LD measures in Table 1. The promoter haplotypes were reconstructed statistically from the population genotype data by using the PHASE program with the Markov chain method for haplotype assignments [25]. A set of logistic regression analyses was performed to examine the association between the *IL-6 *genotypes and symptomatic DIP OA. Crude and adjusted odds ratios (ORs) and their 95% confidence intervals (CIs) were calculated with the SPSS statistical package (Statistical Package for the Social Sciences, version 14.0; SPSS Inc., Chicago, IL, USA). The ORs were adjusted for age (in years), occupation (dentists versus teachers), BMI (tertiles) and smoking history (never versus some time) as potential confounders. Because the haplotype and genotype analyses that followed the initial allelic associations were not entirely independent tests, the *P *values were not corrected for multiple testing.

**Table 1:** Linkage disequilibrium measures between the studied *IL-6 *promoter single nucleotide polymorphisms

Measure	rs1800797 and rs1800796	rs1800797 and rs1800795	rs1800796 and rs1800795
*D'*	1.0 (0.78–1.0)	0.968 (0.94–0.99)	1.0 (0.78–1.0)
LOD	7.53	184.12	7.79
*r*^2^	0.045	0.885	0.048
Haplotypes	AG 54.0%	AC 53.3%	GC 55.5%
	GC 3.7%	GC 2.2%	CG 3.7%
	GG 42.3%	GG 43.8%	GG 40.8%
		AG 0.7%	

D' – statistical measure of linkage disequilibrium (D' = 1 is known as complete linkage). The numbers in parentheses indicate 95% confidence intervals for D'. The lod score (LOD) serves as a test of the null hypothesis of free recombination versus the alternative hypothesis of linkage. LOD >3 is traditionally regarded as evidence for linkage.

r2 is a correlation coefficient between pairs of loci.

## Results

### Clinical findings

Of the 535 successfully genotyped participants, 48 (9%) were diagnosed as having symptomatic OA in at least two DIP joints. The basic characteristics of the subjects by OA status are presented in Table 2.

**Table 2:** Characteristics of the material

Characteristic	Total	Symptomatic DIP OA		*P*^a^
			
		No	Yes	
Number (percentage)	535 (100)	487 (91.0)	48 (9.0)	
Occupation				0.068
Dentists, *n *(percentage)	290 (54.2)	270 (55.4)	20 (41.7)	
Teachers, *n *(percentage)	245 (45.8)	217 (44.6)	28 (58.3)	
Age, years (mean ± SD)	53.9 ± 5.3	53.6 ± 5.2	57.7 ± 4.2	0.0001
BMI (mean ± SD)	24.5 ± 3.6	24.4 ± 3.6	25.0 ± 3.3	0.24
Smoking history				0.12
Never, *n *(percentage)	396 (74.0)	356 (73.1)	40 (83.3)	
Some time, *n *(percentage)	139 (26.0)	131 (26.9)	8 (16.7)	

BMI, body mass index; DIP OA, distal interphalangeal osteoarthritis. Symptomatic DIP OA was defined by the presence of both radiographic findings of grade 2 or more and symptoms in at least two DIP joints.

^a^*P *value for the comparison between DIP OA status groups (Student's t-test or chi-square test).

### Genetic findings

All the genotype frequencies analyzed were in Hardy–Weinberg equilibrium. No statistically significant differences in the frequencies of the genotypes or carriage rates of the *IL-6 *polymorphisms were observed between the two occupational groups. Because the two occupational groups proved to be homogeneous with regard to the polymorphisms of interest, they were pooled and the results presented here apply to the whole series, except that the statistical calculations have been adjusted for occupation.

No association was observed between DIP OA (radiographic OA) and *IL-6 *promoter polymorphisms, but there was a statistically significant association between the G alleles at promoter positions -174 and -597 and symptomatic DIP OA (Table 3), the G allele being seen more frequently among the subjects with symptomatic DIP OA (*P *= 0.010, *P*_corr _= 0.020 and *P *= 0.012, *P*_corr _= 0.024, respectively). This difference was also evident when comparing the carriers of the G allele at polymorphic locations G-174C and G-597A, in that 67.6% (*n *= 328) of the subjects without symptomatic DIP OA had at least one G (-174) allele in comparison with 87.5% (*n *= 42) of the subjects with the disease (*P *= 0.004), the corresponding figures for the G (-597) allele being 68.2% (*n *= 331) and 87.2% (*n *= 41), respectively (*P *= 0.007). There were no differences between the groups in allele frequencies or carriage rates at the promoter location -572. At G-174C the combined GG and GC genotypes increased the risk of the disease in comparison with the CC genotype (*P *= 0.008), and similar results were obtained for the G-597A polymorphism (*P *= 0.006). The genotypes at the G-572C polymorphic location had no effect on the risk of symptomatic OA.

**Table 3:** Frequency of the *IL-6 *(G-174C, G-572C, G-597A) genotypes, by DIP OA status

Genotype	Symptomatic DIP OA				*P*^c^
		
	Condition absent (*n *= 485)		Condition present (*n *= 47–48)		
	*n*	Percentage	*n*	Percentage	
*IL-6*(G-174C)^a^					0.016
GG	93	19.2	13	27.1	
GC	235	48.5	29	60.4	
CC	157	32.4	6	12.5	
G allele carriage	328	67.6	42	87.5	0.004
G allele frequency	421	43.4	55	57.3	0.010^d^
*IL-6*(G-572C)^b^					0.362
GG	451	93.0	42	89.4	
GC	34	7.0	5	10.6	
C allele carriage	34	7.0	5	10.6	0.362
C allele frequency	34	3.5	5	5.3	0.382
*IL-6*(G-597A)^a^					0.023
GG	103	21.2	14	29.8	
GA	226	47.0	27	57.4	
AA	154	31.8	6	12.8	
G allele carriage	331	68.2	41	87.2	0.007
G allele frequency	432	44.5	55	58.5	0.012^e^

DIP OA, distal interphalangeal osteoarthritis. Symptomatic DIP OA was defined by the presence of both radiographic findings of grade 2 or more and symptoms in at least two DIP joints.

^a^Genotypes were available for 533 subjects; ^b^Genotypes were available for 532 subjects. ^c^*P *value is given for the comparison between DIP OA status groups (Chi-square test or Fisher's exact probability test); ^d^*P*_corr _= 0.02, odds ratio 1.75 (95% confidence interval 1.15 to 2.67); ^e^*P*_corr _= 0.024, odds ratio 1.76 (95% confidence interval 1.15 to 2.69).

The three *IL-6 *promoter polymorphisms revealed a total of five haplotypes. The most common was C-G-A (0.53), followed by G-G-G (0.40), whereas the others occurred at a combined frequency of only 0.07 (data not shown). No statistically significant associations of a haplotype containing the G allele at each locus with DIP OA and symmetrical DIP OA were observed (OR 1.24, 95% CI 0.85 to 1.83, and OR 1.42, 95% CI 0.96 to 2.10; Table 4), whereas the haplotype G-G-G was overrepresented in the women with symptomatic DIP OA in comparison with those without the disease (0.51 versus 0.39, *P *= 0.023). Analysis of the G-G-G haplotype pairs (diplotypes) showed that the G-G-G/other diplotype was overrepresented among the women with symptomatic DIP OA (70.8% versus 45.8%, *P *= 0.001; Table 4). The risk of symptomatic DIP OA was increased in the carriers of the G-G-G haplotype (OR 4.45, 95% CI 1.82 to 10.88).

**Table 4:** Frequency of the *IL-6 *G-G-G diplotypes, by the DIP OA status (*n *= 533 to 535)

Condition	Diplotypes	Condition absent		Condition present		Odds ratio (95% confidence interval)	
		*n*	Percentage	*n*	Percentage	Crude	Adjusted
DIP OA							
Total *n*		309		224			
	other/other	117	37.9	74	33.0	1.00	1.00
	G-G-G/other	148	47.9	107	47.8	1.14 (0.78–1.68)	1.17 (0.77–1.76)
	G-G-G/G-G-G	44	14.2	43	19.2	1.54 (0.93–2.58)	1.48 (0.86–2.55)
	G-G-G carriage	192	62.1	150	67.0	1.23 (0.86–1.77)	1.24 (0.85–1.83)
	G-G-G frequency	236	38.2	193	43.1	1.22 (0.96–1.57)	1.20 (0.92–1.56)
Symmetrical DIP OA							
Total *n*		329		205			
	other/other	127	38.6	64	31.2	1.00	1.00
	G-G-G/other	155	47.1	101	49.3	1.29 (0.87–1.91)	1.35 (0.89–2.04)
	G-G-G/G-G-G	47	14.3	40	19.5	1.69 (1.01–2.83)	1.65 (0.95–2.84)
	G-G-G carriage	202	61.4	141	68.8	1.38 (0.96–2.00)	1.42 (0.96–2.10)
	G-G-G frequency	229	35.9	181	42.1	1.30 (1.01–1.67)^a^	1.29 (0.99–1.68)
Symptomatic DIP OA							
Total *n*		487		48			
	other/other	185	38.0	6	35.7	1.00	1.00
	G-G-G/other	223	45.8	34	70.8	4.70 (1.93–11.44)^a^	5.03 (2.02–12.51)^a^
	G-G-G/G-G-G	79	16.2	8	16.7	3.12 (1.05–9.29)	3.01 (0.98–9.22)
	G-G-G carriage	302	62.0	42	87.5	4.29 (1.79–10.28)^a^	4.45 (1.82–10.88)^a^
	G-G-G frequency	381	39.1	50	51.0	1.83 (1.19–2.81)^a^	1.62 (1.07–2.45)^a^

Distal interphalangeal osteoarthritis (DIP OA) was defined by the presence of radiographic findings of grade 2 or more in at least two DIP joints. Symmetrical DIP OA was defined by the presence of radiographic findings of grade 2 or more in at least one symmetrical pair of the DIP joints. Symptomatic DIP OA was defined by the presence of both radiographic findings of grade 2 or more and symptoms in at least two DIP joints.

^a^*P *value remained statistically significant after correcting for multiple testing.

In addition, when the diplotypes for the -174 and -597 loci were analyzed together, the findings indicated that carriage of the G-G diplotype increased the risk of both symmetrical DIP OA (OR 1.52, 95% CI 1.01 to 2.28) and symptomatic DIP OA (OR 3.67, 95% CI 1.50 to 9.00; Table 5), although only the statistically significant association between the G-G diplotype and symptomatic DIP OA remained after correcting for multiple testing.

**Table 5:** Frequency of *IL-6 *G-G diplotypes (G-597A; G-174C) by DIP OA status (*n *= 528 to 530)

Condition	Diplotypes	Condition absent		Condition present		Odds ratio (95% confidence interval)	
		*n*	Percentage	*n*	Percentage	Crude	Adjusted
DIP OA							
Total *n*		306		222			
	other/other	105	34.3	64	28.8	1.00	1.00
	G-G/other	147	48.0	109	49.1	1.22 (0.82–1.81)	1.27 (0.83–1.95)
	G-G/G-G	54	17.6	49	22.1	1.49 (0.91–2.44)	1.42 (0.84–2.41)
	G-G carriage	192	62.1	150	67.0	1.29 (0.89–1.87)	1.31 (0.88–1.96)
	G-G frequency	237	39.9	207	44.8	1.22 (0.96–1.56)	1.19 (0.92–1.55)
Symmetrical DIP OA							
Total *n*		325		204			
	other/other	114	35.1	55	27.0	1.00	1.00
	G-G/other	155	47.7	102	50.0	1.36 (0.91–2.05)	1.45 (0.94–2.23)
	G-G/G-G	56	17.2	47	23.0	1.74 (1.05–2.88)	1.69 (0.99–2.87)
	G-G carriage	302	62.0	42	87.5	1.46 (1.00–2.15)	1.52 (1.01–2.28)
	G-G frequency	267	41.1	196	48.0	1.33 (1.03–1.70)	1.31 (1.01–1.71)
Symptomatic DIP OA							
Total *n*		483		47			
	other/other	163	33.7	6	12.8	1.00	1.00
	G-G/other	230	47.6	28	59.6	3.31 (1.34–8.17)^a^	3.62 (1.43–9.15)^a^
	G-G/G-G	90	18.8	13	27.7	3.92 (1.44–10.68)^a^	3.79 (1.35–10.61)^a^
	G-G carriage	302	62.0	42	87.5	3.48 (1.45–8.37)^a^	3.67 (1.50–9.00)^a^
	G-G frequency	410	42.4	54	57.4	1.83 (1.19–2.81)^a^	1.62 (1.07–2.45)

Distal interphalangeal osteoarthritis (DIP OA) was defined by the presence of radiographic findings of grade 2 or more in at least two DIP joints. Symmetrical DIP OA was defined by the presence of radiographic findings of grade 2 or more in at least one symmetrical pair of the DIP joints. Symptomatic DIP OA was defined by the presence of both radiographic findings of grade 2 or more and symptoms in at least two DIP joints.

^a^*P *value remained statistically significant after correcting for multiple testing.

## Discussion

The present study showed an association between certain promoter genotypes of *IL-6 *and the more severe outcomes of DIP OA, namely symmetrical and symptomatic DIP OA. IL-6 is one of the most important mediators of inflammatory reactions in humans. At least 50 SNPs and five common haplotypes have been identified so far in the *IL-6 *gene, and the genetic variations, especially within the non-coding promoter sequence, have been shown to have a powerful influence on the expression of the gene [26-28]. Pain and inflammation symptoms are known to be related to IL-6, and it was recently reported by Oen and coworkers that the promoter genotype -174GG has a positive correlation with pain in juvenile rheumatoid arthritis [19].

It has been reported previously that IL-6 production *ex vivo *is greater in individuals who are homozygous for the haplotype containing G at -597 and -174 [29]. This is interesting in the light of the fact that both of these G alleles substantially increased the risk of symptomatic OA in our material. Individually, the G allele of the G-174C variation has repeatedly been shown to associate with increased expression and plasma levels of IL-6 [17,30], and the same polymorphism has recently been directly linked to hip OA, because the CC genotype was significantly higher in the control population [18]. The three promoter variations G-597A, G-572C and G-174C have been shown to influence *IL-6 *transcription through a complex interaction determined by the haplotype, and the G alleles at these loci have been found to associate with increased transcription of *IL-6 *[16]. Our results strongly support this finding, because the G-G-G haplotype was clearly overrepresented among those with a symptomatic disease in our sample.

OA can be defined by symptoms or pathology (radiographic features). Although osteoarthritis is regarded as a likely origin of joint pain [31], the association between radiographic evidence of OA and symptoms in the general population seems to be rather poor, as many persons with radiographic OA do not have any symptoms, and vice versa [32]. The American College of Rheumatology criteria for the classification of OA identify cases of persistent pain (most days for at least 1 month). The proportion of radiographic OA that is symptomatic has been estimated to be between 20% and 40%, and most persons with radiographic OA do not have persistent symptoms [32]. Although the American College of Rheumatology criteria are the most frequently used definition of symptomatic hand OA for clinical studies, their limitations for epidemiological studies have been recognized [32]. The available evidence suggests that radiography is better than clinical examination for defining hand OA in epidemiological studies, and that it is possible to identify persons with clinically significant OA by combining a radiographic criterion with self-reported symptoms [32]. It has recently been shown in this same population that the severity of finger joint pain is clearly dependent on the severity of radiographic OA [33].

We sought here to examine more severe cases of OA, those that are more likely to have a genetic component. All the subjects were uniformly assessed for the presence of radiographic DIP OA and the joint-specific occurrence of symptoms. The outcome of symptomatic DIP OA in at least two joints was chosen in view of its assumed clinical relevance and its specificity in terms of joint location.

Although our results are supported by the findings of several functional studies of *IL-6 *gene transcription [15,16,28,29,34], there are also negative results concerning the contribution of promoter variability [35]. One explanation for this could be the effect of aging, which may overwhelm the genetic effect on the IL-6 levels. There is substantial evidence that the increase in IL-6 serum levels with age results in part from the loss of sex steroids such as estrogen, testosterone and dehydroepiandrosterone [36,37], which have an important role in blocking transcription of the *IL-6 *gene, so that their loss at menopause may have a more conspicuous role than the genotype.

Our findings do not suggest an association between the development of asymptomatic DIP OA and the *IL-6 *gene, because the risk of radiographic OA was not affected by the *IL-6 *genotype. The number of individuals with radiographic DIP OA having symptoms turned out to be relatively low compared with the total number of subjects, which reduced the power of the results, so that replication with a larger sample would be beneficial. In addition, a cross-sectional study setting may result in difficulties in accurately estimating the true risk of DIP OA associated with *IL-6 *promoter variants. The experiencing and evaluation of symptoms, particularly pain, are always individual, purely subjective and likely to fluctuate with time. It should be noted that a self-administered questionnaire cannot preclude other causes of joint pain in addition to DIP OA. In contrast, asymptomatic periods of variable duration are typical of osteoarthritis, and the present subjects were prompted to report symptoms that had occurred within the previous 30 days. It is therefore possible that the number of symptomatic subjects in our sample is lower than it should be, as a result of the exclusion of those who were going through an asymptomatic period at the time of answering the questionnaire. The association between *IL-6 *and symptomatic DIP OA may therefore be even stronger than that reported here. A strict time scale for the occurrence of reported symptoms combined with the radiological analysis should reduce the amount of bias caused by temporary, indistinct joint symptoms not caused by DIP OA. In general, this work underlines the importance of a homogeneous study population with a specific outcome formulation, to avoid allowing the modest genetic contribution to be overwhelmed by the clinical diversity of the subjects.

## Conclusion

Our findings lend support to the notion of an association between promoter variations in the *IL-6 *gene and symptomatic and symmetrical DIP OA, outcomes that can be presumed to be of high clinical relevance. It may be possible in future to make therapeutic use of the knowledge of IL-6 and its significance as a cause of inflammation and pain, in treating symptoms of arthritis. Specific IL-6 receptor antagonists inhibiting the inflammation cascade within the articular cartilage are a relevant option when designing new therapeutic interventions for this disease.

## Abbreviations

BMI = body mass index; CI = confidence interval; DIP = distal interphalangeal; IL-6 = interleukin-6; LD = linkage disequilibrium; OA = osteoarthritis; OR = odds ratio; SNP = single nucleotide polymorphism.

## Competing interests

The authors declare that they have no competing interests.

## Authors' contributions

OK conducted the molecular genetic studies and drafted the manuscript. SS participated in the design of the study, performed the statistical analysis and participated in writing the manuscript. TV and KL conducted the radiological assessment. HR, LA, MM and PL participated in the design and coordination of this study and participated in writing the manuscript. All authors read and approved the final manuscript.
